# Examining nurses’ attitudes toward patients who use substances in the hospital setting: A scoping review

**DOI:** 10.1016/j.ijnsa.2026.100513

**Published:** 2026-02-24

**Authors:** Andrea Raynak, France Paquet, Amanda Bakke, Brianne Wood, Michel Bédard, Christopher Mushquash, Debra Gold, Hunter Polonoski

**Affiliations:** a Director, Nursing Practice & Learning, Thunder Bay Regional Health Sciences Centre, Thunder Bay, ON, P7B 6V4, Canada; b Chief Nursing Executive, Vice President Professional Practice, 1111 Ghislain Street, Hawkesbury, ON K6A 3G5, Canada; c Research Assistant, Thunder Bay Regional Health Sciences Centre, Thunder Bay, ON, P7B 6V4, Canada; d Associate Scientist, Thunder Bay Regional Health Sciences Centre, Thunder Bay, ON, P7B 6V4, Canada; e Centre for Social Accountability, NOSM University, Thunder Bay, ON, P7B 5E1, Canada; f Professor, Department of Health Sciences, Member, Centre for Research on Safe Driving, Lakehead University, Canada; g Scientific Director, Centre for Applied Health Research, St. Joseph's Care Group, 955 Oliver Road, Thunder Bay, ON, P7B 5E1, Canada; h Canada Research Chair in Indigenous Mental Health and Addiction, Professor, Department of Psychology, Lakehead University, Thunder Bay, ON, Canada; i Professor, Northern Ontario School of Medicine University, Thunder Bay, ON, Canada; j Psychologist, Dilico Anishinabek Family Care, Fort William First Nation, ON, Canada; k Vice President Research, Thunder Bay Regional Health Sciences Centre, Canada; l Chief Scientist, Thunder Bay Regional Health Research Institute, Canada; m Thunder Bay Regional Health Sciences Centre, Thunder Bay, ON, P7B 6V4, Canada; n Liaison Librarian for Health Sciences, Nursing & Sociology, Lakehead University, Thunder Bay, ON P7B 5E1, Canada

**Keywords:** Nurses’ attitudes, Substance use, Hospital care, Scoping review

## Abstract

**Aim:**

This scoping review aimed to map and describe the existing literature on nurses’ attitudes toward hospitalized patients who use substances (PWUS).

**Design:**

A scoping review was conducted in accordance with Joanna Briggs Institute (JBI) methodology and reported using the PRISMA-ScR guidelines.

**Methods:**

A systematic search was conducted on August 27, 2024, in PubMed, PsycINFO, and CINAHL to identify peer-reviewed studies published in English, between 2014 and 2024. Eligible studies included original qualitative and quantitative research examining nurses’ attitudes toward PWUS in hospital settings. Two independent reviewers conducted study selection using a structured screening process. Data were extracted using an author-developed tool capturing study characteristics, including country of origin, aims, methodologies, nursing context, hospital setting, approaches to assessing attitudes, and key findings. Extracted data were synthesized descriptively to map study characteristics, conceptual patterns, and thematic emphases within the literature. Of 1568 abstracts screened, 13 studies met inclusion criteria, with four additional studies identified through citation mining, resulting in 17 included articles.

**Results:**

The included literature predominantly described negative attitudes toward PWUS among nurses in hospital settings, alongside recurring thematic emphases related to substance use knowledge and education, challenges in pain management, and perceptions of limited organizational support. Studies varied widely in design, measurement approaches, and geographic context. Findings were organized using Bronfenbrenner’s socioecological model to illustrate how nurses’ attitudes have been examined across individual, interpersonal, organizational, and societal levels, with most studies concentrated at the micro- and mesolevels.

**Conclusion:**

This scoping review maps a growing but methodologically heterogeneous body of literature examining nurses’ attitudes toward PWUS in hospital care. The findings highlight conceptual, methodological, and contextual gaps in the existing evidence, particularly related to organizational and macrolevel influences. These gaps highlight important directions for future research aimed at advancing a more comprehensive understanding of nurses’ attitudes within hospital-based substance use care.

**What is already known:**

• Nurses demonstrate negative attitudes towards patients who use substances in the hospital setting

• Patient care is affected by nurses negative attitudes

• Nurses have advocated for more support in caring for this patient population.

Alt-text: Unlabelled box dummy alt text

**What this paper adds:**

• Nurses’ attitudes toward hospitalized patients who use substances are frequently negative, with challenges noted in pain management, substance use knowledge, and perceptions of limited organizational support.

• Existing research is heterogeneous in design, measurement, and geographic scope, with most studies focusing on individual and interpersonal factors, leaving organizational and societal influences underexplored.

• By mapping current evidence and identifying recurring themes and gaps, this review provides a framework to inform future nursing education, organizational strategies, and policy development to improve care for patients who use substances.

Alt-text: Unlabelled box dummy alt text

## 1 Introduction

Substance use is a significant contributor to hospital admissions and is associated with a range of adverse inpatient outcomes, including infections, overdose, premature discharge, readmission, and increased mortality (Strike et al., 2020; Wright et al., 2020). Hospitalized patients who use substances (PWUS) often experience complex clinical and psychosocial needs, particularly related to pain management (Mayer et al., 2025; Sowicz et al., 2022), withdrawal (Simon et al., 2020), and stigma (Christian et al., 2025; Mayer et al., 2023; Webster et al., 2025). These challenges contribute to poorer patient experiences (Mayer et al., 2023) compromised care trajectories (Compton et al., 2021), and increased healthcare utilization (Compton et al., 2021;Tan et al., 2020).

Within hospital settings, nurses play a central role in the care of PWUS, as they are primarily responsible for symptom assessment, pain and withdrawal management, ongoing monitoring, and the development of therapeutic relationships. However, hospitalized PWUS may engage in high-risk behaviours such as sharing injection equipment or using contaminated substances, particularly when withdrawal symptoms, pain, or emotional distress are inadequately managed (Dong et al., 2020; Strike et al., 2020). These behaviours increase the risk of complications such as blood-borne infections, soft tissue infections, and overdose, further complicating inpatient care and recovery (Wright et al., 2020).

Among the most distressing experiences reported by PWUS is the inadequate or denied administration of pain medication (Compton et al., 2021; Grant et al., 2025; Mayer et al., 2025; Sowicz et al., 2022). Patients have described not being believed, having their pain underestimated, and being undermedicated due to assumptions of drug-seeking behaviour (Christian et al., 2025; Horner et al., 2019; Strike et al., 2020). These experiences have been associated with avoidance of medical care, delayed presentation, and poorer health outcomes (Christian et al., 2025; Carusone et al., 2019; Strike et al., 2020). In many cases, patients leave hospital before completing treatment, commonly referred to as leaving against medical advice, resulting in missed opportunities for care (Simon, Snow and Wakeman, 2020; Strike et al., 2020). Leaving against medical advice has been linked to lower follow-up rates, increased likelihood of future premature discharges, higher readmission rates within two weeks, and a twofold increase in mortality (Allen et al., 2020; Hyshka et al., 2019). These outcomes also carry substantial financial implications; a national study by Tan et al. (2020) found that hospital readmissions following against medical advice discharges accounted for more than 400,000 inpatient days and an estimated $822 million in healthcare expenditures.

The COVID-19 pandemic further intensified challenges within nursing practice, including increased patient acuity, staffing shortages, and worsening working conditions (Lake et al., 2024; de Vos et al., 2024). These pressures have strained the profession and may disproportionately affect interactions with vulnerable populations, including PWUS in hospital settings (Al-Awadhi et al., 2017; Yaghmour, 2022). Research has consistently demonstrated that nurses report more negative attitudes toward PWUS compared to patients with other health conditions (Mulyani et al., 2021), which may influence care delivery, communication, and clinical decision-making.

Although many nurses view the care of PWUS as meaningful and aligned with professional values, the literature also documents experiences of low motivation, emotional exhaustion, and dissatisfaction when working with this population (Johansson and Wiklund‐Gustin, 2016; Kiepek et al., 2021; Mahmoud et al., 2021; Van Boekel et al., 2013). Perceptions of PWUS as difficult, disruptive, or unsafe may contribute to discomfort, heightened safety concerns, and more authoritarian or task-focused approaches to care, undermining therapeutic relationships (Antill Keener et al., 2023; Hakala et al., 2020; Menard-Kocik and Caine, 2021). As a result, patients may withhold information about substance use, complicating assessments and limiting opportunities for meaningful engagement and intervention (Atashzadeh-Shoorideh et al., 2020; Renbarger et al., 2021).

Given the central role of nurses in shaping inpatient experiences and outcomes for PWUS, a comprehensive synthesis of the literature examining nurses’ attitudes toward this population is warranted. Although recent reviews have examined mental health nurses’ perceptions (Merrick et al., 2022), emergency and general health care providers’ attitudes (Cazalis et al., 2023), and interventions addressing barriers to care (Magnan et al., 2024), no review has specifically synthesized evidence on hospital-based nurses’ attitudes toward patients who use substances across diverse clinical settings. A scoping review was selected because it allows for the mapping of heterogeneous evidence, including qualitative and quantitative studies, without focusing on study quality or intervention effectiveness (Peters et al., 2020). This methodology is well suited to identify knowledge gaps, clarify concepts, and highlight areas for future research. To the researchers’ knowledge, no recent review has exclusively examined nurses’ perspectives in this context. Therefore, this scoping review aimed to map the extent, nature, and characteristics of the literature on nurses’ attitudes toward patients who use substances in hospital settings and to identify conceptual and methodological gaps to guide future research.

## 2 Method

This scoping review followed the Joanna Briggs Institute methodology (Aromataris et al., 2024) and adhered to PRISMA-ScR reporting guidelines (see Appendix A; Tricco et al., 2018). A review protocol was not developed prior to conducting this. Grey literature and secondary research (e.g., scoping reviews, systematic reviews, and meta-analyses) were not included. While the Joanna Briggs Institute guidance allows for their inclusion to capture a broad range of evidence, this review focused on primary, peer-reviewed studies to maintain methodological consistency, manage feasibility, and synthesize original findings directly reported by study authors (Peters et al., 2020). Limiting the review in this way allowed for a transparent synthesis of evidence relevant to nurses’ attitudes toward patients who use substances in hospital settings.

For this review, nurses included registered nurses (RNs) and registered practical nurses (RPNs), excluding other hospital providers because of their limited time interactions with this patient population. The term substance encompassed alcohol and drug use, excluding tobacco and cannabis (see Table 1). Drug use was defined as the use of psychoactive substances that may lead to dependence, withdrawal, or other health-related complications, including illicit drugs (e.g., opioids, stimulants, cocaine, methamphetamines) and the non-medical or problematic use of prescription medications (e.g., opioids, benzodiazepines) (Mclellan, 2017). This review focused on the clinical challenges and behaviors associated with substance use disorders, rather than occasional or medically supervised use of prescription drugs. Tobacco and cannabis were excluded not because they are harmless, but because hospital admissions related to these substances generally do not present the same acute behavioral or care complexities as alcohol and other drugs and therefore warrant separate investigation.

**Table 1:** Inclusion and exclusion criteria.

	Inclusion criteria	Exclusion criteria
Focus	Studies on nurses’ attitudes toward PWUS in the hospital setting	Any other topic
Substance	Drug(s) and/or alcohol	Tobacco, marijuana
Context	Acute care hospital setting	Other settings (e.g., community, outpatient)
Population	Nurses (i.e., RNs, RPNs)	Other health professionals (i.e., physicians, residents, social workers, etc.)
Language	English	Other languages

**Note:** Only studies that fully met all inclusion criteria were included. Studies including tobacco or marijuana, non-hospital settings, or populations other than nurses were excluded. For studies with mixed healthcare providers or inpatient/outpatient settings, data were not extracted unless hospital-based nurse results were clearly reported. Emergency departments were considered part of the acute care hospital context.

Comprehensive searches were conducted on August 27, 2024, across the following databases: PubMed, PsycINFO (ProQuest), and CINAHL (EBSCO). The search was restricted to original research articles published between 2014 and 2024 that had been peer reviewed and written in English. This timeframe for the review was chosen to reflect the evolving landscape of substance use in the hospital setting. Over the past decade, the incidence of substance use has risen significantly, coinciding with the expansion of harm reduction initiatives such as naloxone distribution and supervised consumption services. These developments have likely shaped nurses’ attitudes and clinical practices, making this period particularly relevant for investigation.

The initial search strategy was developed with a medical librarian in PubMed and then adapted for the other databases. Subject headings were modified slightly according to the repository in each database (see Appendix B). Search results were uploaded into Rayyan, an intelligent systematic review software for reference management (Ouzzani et al., 2016). After duplicates were removed, abstracts were screened against the inclusion criteria, and the remaining articles were then reviewed in full, with exclusions made as necessary by the primary researcher (A.R.). This process was repeated independently by the second researcher (F.P.). Once both researchers completed their reviews, results were compared, and reviewers’ disagreements were resolved through consensus. In addition, citation mining was conducted by reviewing the reference lists of included articles to identify additional relevant studies.

Data were independently extracted from each included study by two reviewers (A.R. & F.P.), with a research assistant (A.B.) providing support. Both reviewers extracted data from all articles separately, and discrepancies were resolved through discussion to ensure accuracy and reproducibility using a customized data extraction tool developed specifically for this review (see Table 2). The initial version of the data extraction tool was refined and revised as needed during the extraction process. The extracted data included key study details: country of origin, study aim/purpose, methodology, nursing area of work, hospital description, tools/approaches used to assess attitudes, and study outcomes. In line with the objectives of this scoping review, namely, to map the breadth and depth of the topic, and considering the inclusion of diverse study designs, a formal quality appraisal was not performed. This review was not registered. Lastly, some studies included in this review used non-person-centred terms such as *substance abuse* or *alcohol abuse*. For the purposes of this review, these terms were replaced with more person-centred language such as *substance use* or *substance use difficulty* to reduce stigma and reflect the complexity of substance-related challenges more accurately.

**Table 2:** Data extraction.

Author(s); Yr of Publication; Journal	Country of origin	Aims/Purpose methodology	Study	Population; area of work; hospital description	Tools/approach to examine nurses attitudes	Outcome(s)
Babiarczyk et al. (2024); *Nursing in the 21st Century*	Podbeskidzie, Poland	Assess attitudes of nurses caring for Alcohol Use Disorder (AUD) patients.	Descriptive – cross sectional, quantitative	120, all units, tertiary hospital	Questionnaire	More frequent contact with AUD patients = more aggression toward patients. Majority agreed with statements categorizing AUD patients as “blocking beds”, “rude and aggressive”, responsible for their health problems and less cooperative. Length of time worked decreased agreement with these statements. Most frequent feelings = resentment, anger and fear.
Chozom et al. (2021); *Journal of Nursing Practice*	Bhutan	Explore the prevailing attitudes of nurses towards AUD patients, and to further explore the factors influencing these attitudes.	Thematic analysis, qualitative	15, all units, large hospital in country’s capital	Interviews	4 themes: Attribution beliefs (belief that alcohol use is due to problems in life), Providing care (nurses enjoy helping AUD patients in their time of need), Factors influencing attitudes (readmissions, lack of knowledge, challenges changing patients views, aggressive behaviour), Experience (senior staff better with patients).
Author(s); Yr of Publication; Journal	Country of origin	Aims/Purpose methodology	Study	Population; area of work; hospital description	Tools/approach to examine nurses attitudes	Outcome(s)
Hakala et al. (2020); *Journal od Addictions Nursing*	Finland	Describe nurses' skills, knowledge of care, and attitudes toward the care of patients with alcohol intoxication.	Content analysis - qualitative	6, sobering unit in ED, central hospital	Interviews	5 themes: Skills to discuss alcohol use (majority nurses occasionally or never asked about alcohol use), Safety skills (experiences of violent behaviour), Teamwork skills (collaboration with other nurses and physicians important), Skills organizing follow-up (majority said difficult to arrange), Attitudes (difficult caring for patients, more education needed, seeing alcohol use as illness improved attitudes)
Horner et at. (2019), *PLOS ONE*	Boston MA, USA	Assess the attitudes, perceptions, and training needs of nurses in the inpatient setting when caring for patients who have opioid use disorder.	Thematic analysis	22, all units, large urban academic medical centre	Interviews	6 themes: Stigma (acknowledgement that this is prevalent and impacts the healthcare received), Safety /Security (personal safety concerns of female nurses–rely on security), Assessing / treating pain (nurses had issues believing pain and feeling like they are elevating the problem), Communication (positive between providers), Burnout (common with this patient base), Opportunities for change (standardized care, emotional support and education.)
Author(s); Yr of Publication; Journal	Country of origin	Aims/Purpose methodology	Study	Population; area of work; hospital description	Tools/approach to examine nurses attitudes	Outcome(s)
Hyde et al. (2024); *Journal of Addictions Nursing*	Alberta, Canada	Explore the knowledge, attitudes, and perceptions of acute care nurses caring for patients with AUD.	Descriptive - cross-sectional, exploratory	93, inpatient medicine (7 units) and acute care (6 units), large hospitals over 5 geographic areas	Questionnaire	28 % described working knowledge of AUD, 53 % indicated interest in understanding AUD, 30 % stated they would want to work with AUD patients. More education about AUD patients significantly increased feelings of knowledge about AUD and satisfaction about the care nurses give to AUD patients.
Johansson & Wiklund-Gustin (2015), *Scandinavian Journal of Caring Sciences*	Sweden	Describe how nurses’ working in inpatient psychiatric care experience caring encounters with patients suffering from substance use disorder (SUD).	Qualitative content analysis	6, psychiatric unit, psychiatric hospital	Reflective dialogues	4 themes: Balance between understanding and frustration, Being supportive while maintaining order, Remaining observant of problems while focusing on health of patients, Caring for patients while thinking of one’s own safety. Common theme: Multifaceted vigilance.
Author(s); Yr of Publication; Journal	Country of origin	Aims/Purpose methodology	Study	Population; area of work; hospital description	Tools/approach to examine nurses attitudes	Outcome(s)
Keener et al. (2023); *Journal of Addictions Nursing*	Appalachian Mountains region, USA	Describe the perceptions of nurses who provided care for patients with substance use disorder (SUD).	Descriptive – cross sectional, content analysis	488, all units, large academic medical centre	Questionnaire	Challenges identified: managing pain of patients with SUD, safety, lack of collaboration, distrust. Emotional responses: feeling defeated/burnt out, difficulty showing compassion when patients incompliant, patients described as manipulative/demanding Resources Needed: education, pain scales, designated units, patient liaisons, and community resources.
Author(s); Yr of Publication; Journal	Country of origin	Aims/Purpose methodology	Study	Population; area of work; hospital description	Tools/approach to examine nurses attitudes	Outcome(s)
Kratovil et al. (2023); *American Journal of Nursing*	USA	Explore hospital nurses’ self-assessed knowledge and attitudes about caring for patients who use substances.	Observational / cross- sectional, mixed-methods	691, medical–surgical units, ICUs, EDs, menta**l** health units, and mother–baby units, various hospitals recruited though Facebook	Questionnaire	99 % participants indicated the need for additional training for SUD-related knowledge and skills 4 Themes: Unmet needs (resources, training, education), personal experiences inform care (familial SUD influence perceptions of SUD), personal beliefs (majority saw SUD as a choice), judgmental attitudes (towards patients with SUD)
Mahmoud et al. (2021); *Substance Abuse*	Southwestern Pennsylvania, USA	Examine association between nurses’ demographics, personal/ professional attitudes and motivation to care for patients with opioid use problems.	Descriptive - correlational	234, mental health/emergency/ OBGYN/medical-surgical, 4 hospitals	Questionnaire	Factors increasing motivation to work with opioid-use individuals: personal or familial experience with opioid use, familiarity with this issue, previous experience with these patients, and education.Factors decreasing motivation: perceived patients as dangerous, responsible for their situation, and fear of these patients.
Mahmoud et al. (2023); *Substance Abuse*	Southwestern Pennsylvania, USA	Examine the association between nurses’ demographics, personal/professional attitudes and motivation to care for patients with alcohol use problems.	Descriptive – cross-sectional	234, mental health/emergency/OBGYN/medical-surgical, 4 hospitals	Questionnaire	Factors increasing motivation to work with SU individuals: personal or familial experience with SU, knowledge/continuing education about SU, previous experience with this patient base. Factors decreasing motivation: perceived patients as dangerous, fear, holding the individual responsible and viewing SU as a disease.
Author(s); Yr of Publication; Journal	Country of origin	Aims/Purpose methodology	Study	Population; area of work; hospital description	Tools/approach to examine nurses attitudes	Outcome(s)
Menard-Kocik and Caine (2021), *Canadian Journal of Nursing*	Canada	Explore obstetrical nurses’ perspectives toward caring for pregnant women who use illicit substances in a large inner-city hospital in Western Canada.	Thematic content analysis	18, obstetric unit, large inner-city urban hospital	Interviews	4 themes: Services and care (complexity in care for these patients), Stigma and discrimination (negative personal biases identified), Coping mechanisms (internal struggle to provide holistic care identified), Recommendations for practice (continuing education).
Molinea-Mula et al. (2018); *International Journal of Environmental Research and Public Health*	Balearic Islands, Spain	Assess emergency and mental health nurses’ attitudes and perceptions towards alcoholics.	Descriptive – prospective cross observational	167; mental health and emergency, 5 hospitals	Questionnaire	76 % of the nurses considered alcoholics to be ill individuals, however tendency not to feel comfortable working with them. Negative personalattitudes towards alcohol consumption were predominant.
Morgan (2014); *Pain Management Nursing*	USA	Expand knowledge about nurses’ attitudes and interactions with patients with SUD who are in pain.	Grounded theory approach	14, all units, urban public health hospital	Interview – semi-structured with interview guide	3 themes identified: Inadequate responses to pain management, Delayed response to pain management and difficulties with administrative personnel and their attitudes/perceptions in pain management in patients with SUD.
Munoz et al. (2021); *The American Journal of Maternal Child Nursing*	USA	Determine knowledge and attitudes of nurses and ancillary team members about addictive substance use by women during pregnancy and postpartum.	Descriptive -parametric and nonparametric	109, women’s service areas, Magnet community hospital	Questionnaire	Mean total knowledge score 6.8/8, nurses among highest knowledge scores. 80 % agreed mothers who use drugs have challenges and can successfully recover. 55 % agree mothers who use drugs can be good mothers. Less than half agreed they knew enough about SUD.
Author(s); Yr of Publication; Journal	Country of origin	Aims/Purpose methodology	Study	Population; area of work; hospital description	Tools/approach to examine nurses attitudes	Outcome(s)
Neville and Roan (2014); The *Journal of Nursing Administration*	Northeast Corridor, USA	Investigate RN perceptions of caring for hospitalized medical-surgical patients with substance abuse/dependence.	Descriptive – non-experimental	24, medical-surgical/neurological-orthopedic/oncology units, community medical centre	Questionnaire	4 main themes identified: ethical duty of care, negative perceptions of caring for patients with substance abuse/dependence, need for education, and sympathetic concern. Negative perceptions included anger, distrust, and fear for safety.
Nusbaum (2022); *Journal of Nursing Scholarship*	Israel	Characterizing Israeli nurses’ knowledge, attitudes, and perceptions about opioid misuse and their sense of self-efficacy in managing misuse.	Descriptive - cross-sectional	414, all units, various hospitals recruited through facebook	Questionnaire	47 % reported interaction with patient using opioids in past year. 85 % felt they lacked training to manage misuse. 85.5 % said they would readily care for misusers. 40 % had no opinion or agreed that they don’t accept or understand addiction. 75.6 % reported insufficient institutional support. Most of the participants demonstrated low knowledge levels with a total mean score of 63.1 %.
Shaw et al. (2016); *The American Journal of Maternal Child Nursing*	Washington State, USA	Explore nurses’ perceptions of caring for pregnant and parenting women with a history of opioid misuse.	Grounded theory approach	14,8, obstetrics, 2 large urban birthing centres	Semi-structured interviews	4 themes: Needing more knowledge (education on opioid misuse and caring for these patients), Feeling challenged (struggle of providing good care while dealing with their biases), Expressing concern for mothers and infants (safety of the newborns), Knowing the truth (feeling like they don’t receive the whole truth from patients)

*Note*: The original terminology used to describe individuals who use substances has been retained in the data extraction tables to accurately reflect the language used in the source materials. This terminology has not been adapted to the person-centred language employed throughout the manuscript

## 3 Results

**Fig. 1:**
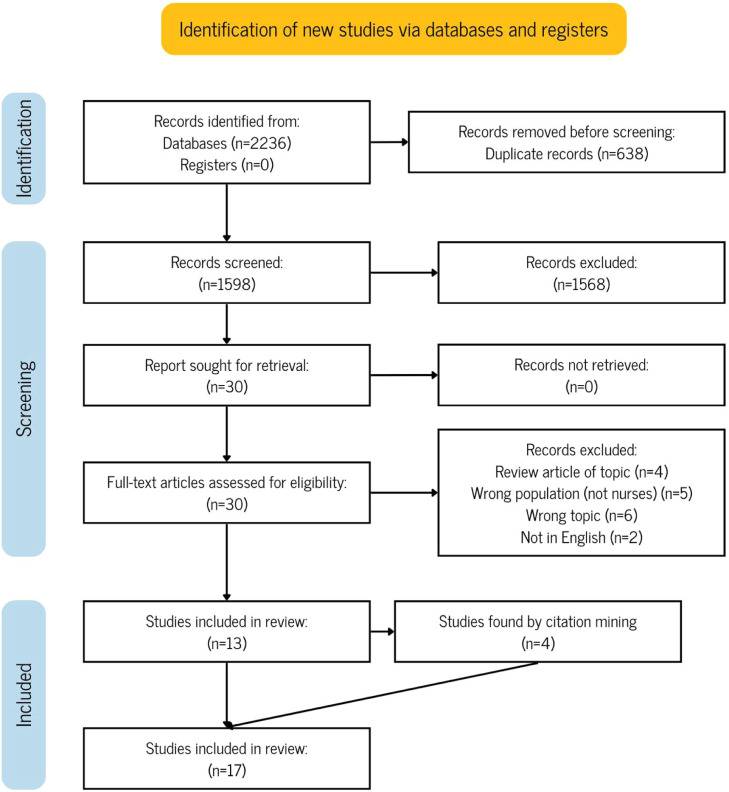
*PRISMA-ScR*.

A total of 1598 articles were identified for screening following the removal of duplicates from the online database search. Screening was conducted based on titles and abstracts, resulting in the exclusion of 1568 articles according to the inclusion and exclusion criteria. The full texts of the remaining 30 articles were retrieved and assessed for eligibility. Of these, 17 articles were excluded for the following reasons: review articles (not original research; *n* = 4), wrong population (*n* = 5), unrelated topic (*n* = 6), and not in English (*n* = 2). This process resulted in 13 full-text articles eligible for inclusion, with an additional four identified through citation mining, bringing the total to 17 articles included in this scoping review (see Fig. 1).

## 4 Study characteristics

### 4.1 Location

Most of the articles in this review originated from the United States (*n* = 9), with additional studies from Canada (*n* = 2), Spain (*n* = 1), Poland (*n* = 1), Finland (*n* = 1), Sweden (*n* = 1), Israel (*n* = 1), and Bhutan (*n* = 1).

### 4.2 Study design

The studies reviewed employed a range of study designs: qualitative (*n* = 8), quantitative (n = 7), or mixed methods (n = 2). Descriptive studies were the most common (n = 8), comprising cross-sectional (n = 5), correlational (n = 1), nonexperimental (n = 1), and those using parametric and nonparametric methods (n = 1). One study integrated cross-sectional, observational, and mixed methods approaches. Among the qualitative studies, grounded theory was used in two studies, and content analysis was applied in five studies.

### 4.3 Tool(s) of measurement

The studies used various methods to measure nurses’ attitudes, including the Seaman-Mannello Scale (*n* = 1), the adapted Alcohol and Alcohol Problems Perceptions Questionnaire (AAPPQ; *n* = 2), self-constructed surveys (*n* = 4), written responses to questions (*n* = 1), semistructured interviews (*n* = 6), the Drug and Drug Problems Perceptions Questionnaire (DDPPQ; *n* = 1), the Survey of Attitudes and Perceptions (*n* = 1), and reflective dialogues (*n* = 1).

### 4.4 Hospital setting

The hospital settings described were varied and included a community medical center, academic medical centers (*n* = 2), a large urban birthing center, an urban public health hospital, an inner-city urban hospital, central hospitals (*n* = 2), a community hospital, and a psychiatric hospital. Notably, seven studies did not provide details about the type or description of their hospital settings.

### 4.5 Inpatient unit

The inpatient units included in the reviewed studies were diverse. Although several studies (*n* = 11) included nurses across all hospital units, others (*n* = 3) focused on specific specialties such as obstetrics/gynecology and mental health units (*n* = 2) and psychiatric units (*n* = 1).

## 5 Themes in the literature

To organize these findings, Bronfenbrenner’s socioecological model (SEM) was applied (Bronfenbrenner, 1979). This model was chosen because it provides a framework for understanding the intricate and dynamic interactions between individual behaviors and external influences that collectively shape the quality of patient care and care outcomes. The SEM was particularly appropriate for this analysis because it allows for the consideration of how nurses’ attitudes and decision-making processes are influenced not only by internal beliefs, experiences, and professional challenges but also by broader structural and societal forces, including institutional policies, workplace environments, and cultural norms (Bronfenbrenner, 1979).

**Fig. 2:**
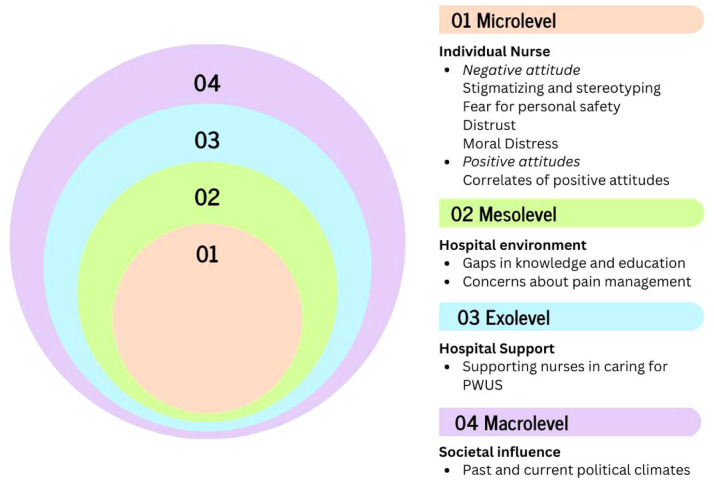
*Application of bronfenbrenner’s socioecological monel to the scoping review findings*.

The SEM conceptualizes these influences across four interrelated levels (Bronfenbrenner, 1979). In this scoping review, they were interpreted as corresponding to the individual nurse (microlevel), including nurse beliefs and attitudes; the hospital environment (mesolevel), including intrapersonal interactions between nurses and PWUS; hospital support (exolevel), including initiatives supporting nurses in caring for PWUS; and societal influence (macrolevel), including past and current political climates. Using this framework facilitates a nuanced understanding of the factors influencing nursing practice (see Fig. 2).

### 5.1 Microlevel: individual nurses’ beliefs and attitudes

#### 5.1.1 Negative attitudes of nurses toward PWUS

##### 5.1.1.1 Stigmatizing and stereotyping

A significant body of literature has underscored the presence of stigmatizing and stereotyping attitudes among nurses toward PWUS (Antill Keener et al., 2023; Babiarczyk et al., 2024; Chozom et al., 2021; Hakala et al., 2020; Horner et al., 2019; Kratovil et al., 2023; Molina-Mula et al., 2018). For instance, Molina-Mula et al. (2018) found that 80.1 % of the nursing participants in their study believed that individuals who consumed alcohol lead unpleasant lives, and 61.9 % associated these patients with poor health outcomes. Similarly, Babiarczyk et al. (2024) reported that the nurses in their study expressed frustration over patients with alcohol use disorder occupying beds intended for more acutely ill patients. Hakala et al. (2020) found that nurses frequently doubted the ability of PWUS to make meaningful lifestyle changes.

Several studies described PWUS as difficult, demanding (Antill Keener et al., 2023; Hakala et al., 2020; Johansson and Wiklund-Gustin, 2016; Morgan, 2014), impulsive, or aggressive (Chozom et al., 2021). Some nurses labeled patients as drug seekers (Morgan, 2014) or perceived them as defensive (Horner et al., 2019). Kratovil et al. (2023) reported that some nurses openly acknowledged judgmental attitudes toward PWUS.

##### 5.1.1.2 Fear for personal safety

Concerns about personal safety were a prominent theme in previous research influencing nurses’ reluctance to care for PWUS (Antill Keener et al., 2023; Hakala et al., 2020; Horner et al., 2019; Menard-Kocik and Caine, 2021; Molina-Mula et al., 2018; Neville and Roan, 2014). Patients with substance use difficulties were described as aggressive, manipulative, and uncooperative (Antill Keener et al., 2023; Babiarczyk et al., 2024; Hakala et al., 2020; Horner et al., 2019; Johansson and Wiklund-Gustin, 2016; Mahmoud et al., 2021, 2023; Neville and Roan, 2014).The fear of potential violence also was identified as contributing to nurse burnout (Antill Keener et al., 2023; Hakala et al., 2020; Horner et al., 2019; Johansson and Wiklund-Gustin, 2016; Neville and Roan, 2014) and heightened concerns over drug diversion, exposure to drug paraphernalia, and the presence of unauthorized visitors (Antill Keener et al., 2023).

To mitigate these risks, nurses employed various strategies, including monitoring signs of patient agitation (e.g., fist clenching, nervousness); carrying personal alarms; and avoiding potential weapons such as scissors (Hakala et al., 2020). Many relied on security personnel to manage high-risk behaviors (Hakala et al., 2020; Horner et al., 2019), further illustrating the significant impact of safety concerns on patient care.

##### 5.1.1.3 Distrust

Another theme in the literature was the perceived lack of honesty among PWUS regarding their substance use, leading to difficulties providing effective care (Hakala et al., 2020; Shaw et al., 2016). Antill Keener et al. (2023) found that inconsistent or false information from patients complicated the ability of nurses to establish and maintain therapeutic relationships.

##### 5.1.1.4 Moral distress

Nurses frequently reported experiencing moral distress when providing care to PWUS, particularly when their ethical duty to offer compassionate care conflicted with internal biases, external stigma, or safety concerns (Antill Keener et al., 2023; Hakala et al., 2020; Munoz et al., 2021; Shaw et al., 2016). In some studies, nurses described feelings of frustration related to patient relapses, ethical dilemmas concerning pain management, and concerns about care in perinatal settings (Chozom et al., 2021; Morgan, 2014; Shaw et al., 2016).

##### 5.1.1.5 Positive attitudes of nurses toward PWUS

Although negative attitudes were prevalent, some researchers highlighted instances of positive attitudes among nurses, particularly in the maternal and neonatal care settings (Menard-Kocik & Caine, 2021; Munoz et al., 2021; Shaw et al., 2016). Nurses caring for pregnant and postpartum women who used substances, as well as infants with neonatal abstinence syndrome, expressed greater compassion and a commitment to providing specialized care. Some studies also reported that nurses expressed positive attitudes toward patients with alcohol use disorders (Chozom et al., 2021; Munoz et al., 2021).

##### 5.1.1.6 Correlates of positive attitudes

Factors associated with more positive attitudes included personal connections to substance use, working in specialized units, higher levels of education, formal substance use training, and cultural norms (Babiarczyk et al., 2024; Chozom et al., 2021; Hyde et al., 2024; Mahmoud et al., 2021, 2023; Munoz et al., 2021). Gender differences were reported in some studies (Molina-Mula et al., 2018; Mahmoud et al., 2023).

### 5.2 Mesolevel: hospital environment, intrapersonal interactions between nurses and PWUS

#### 5.2.1 Gaps in knowledge and education surrounding substance use

Several studies identified gaps in nurses’ knowledge and education on substance use (Antill Keener et al., 2023; Neville and Roan, 2014; Shaw et al., 2016). Studies reported that nurses with more experience or formal training in substance use care reported higher confidence and perceived competence (Chozom et al., 2021; Mahmoud et al., 2023). Despite previous training, some nurses reported needing further education to feel adequately prepared, including knowledge of opioid use in perinatal care and assessment of overdose risk (Menard-Kocik and Caine, 2021; Munoz et al., 2021; Nusbaum and Farkash, 2022).

#### 5.2.2 Nurses’ concerns about managing pain of PWUS

Several studies reported challenges in managing the pain of PWUS in hospital settings (Antill Keener et al., 2023; Horner et al., 2019; Morgan, 2014; Neville and Roan, 2014; Shaw et al., 2016). Nurses described difficulties in assessing pain, patient tolerance to medication, and differentiating legitimate pain from potential drug-seeking behaviors (Morgan, 2014; Neville and Roan, 2014). Concerns regarding opioid use were also reported (Horner et al., 2019; Morgan, 2014). Some studies described educational needs to support nurses in providing appropriate pain management, including for perinatal patients using substances (Shaw et al., 2016).

### 5.3 Exolevel: hospital support, supporting nurses in caring for PWUS

Studies reported that nurses perceived a lack of hospital support for caring for PWUS (Antill Keener et al., 2023; Horner et al., 2019; Kratovil et al., 2023; Morgan, 2014; Nusbaum and Farkash, 2022). Reported gaps included insufficient training, limited resources, and inadequate access to multidisciplinary teams. Some studies described specific resources that nurses identified as helpful, such as pain scales for PWUS, alternative pain management options, specialized units, patient liaisons, and nurse-led protocols (Antill Keener et al., 2023; Morgan, 2014; Kratovil et al., 2023; Nusbaum and Farkash, 2022).

### 5.4 Macrolevel: societal influence on nursing caring for PWUS

Across the included studies, no research explicitly examined macrolevel influences such as sociopolitical context, health policy, criminalization of substance use, or harm reduction ideology as primary analytic variables shaping nurses’ attitudes or care practices toward PWUS. While several studies implicitly referenced broader contextual factors (e.g., substance use stigma, harm reduction principles), these elements were not systematically defined, measured, or analyzed.

## 6 Discussion

To maintain continuity between the Results and Discussion sections, the SEM is used as a conceptual framework to organize and interpret the mapped literature. In keeping with the purpose of a scoping review, this discussion does not advance recommendations for practice or policy; rather, it synthesizes patterns in the existing evidence, highlights conceptual and methodological trends, and identifies gaps that warrant further investigation. Organizing the findings across SEM levels illustrates how nurses’ attitudes toward PWUS have been studied at multiple, interacting levels, while also revealing where the literature remains underdeveloped.

### 6.1 Microlevel: individual nurses’ beliefs and attitudes

This scoping review identified 17 studies conducted across diverse geographic contexts that examined nurses’ attitudes toward PWUS in hospital settings. Across studies, negative attitudes and stigma toward PWUS were commonly reported, mirroring findings documented among other health care professionals, including physicians and social workers (Dhanani and Franz, 2021; Lawrence et al., 2022; Richelle et al., 2022; Temenos et al., 2024). However, the literature also demonstrated heterogeneity in nurses’ responses. Some studies described strong stigmatizing beliefs associated with compromised care quality (Babiarczyk et al., 2024), whereas others reported expressions of empathy, concern, and moral distress despite discomfort in clinical interactions (e.g., Morgan, 2014; Shaw et al., 2016).

Several individual-level factors were examined in relation to nurses’ attitudes, including clinical area of practice, prior exposure to PWUS, personal experiences, and cultural norms (Chozom et al., 2021; Mahmoud et al., 2023; Munoz et al., 2021). Studies conducted in specialized units or addiction-focused contexts more frequently reported nuanced or empathetic perspectives, suggesting that familiarity and experience may influence attitudinal orientation. Methodologically, most studies relied on cross-sectional survey designs or qualitative interviews, limiting insight into how attitudes develop or change over time.

Notably, important gaps were identified at this level. Few studies explicitly examined the role of contextual stressors such as workload, moral distress, or nurse burnout in shaping attitudes toward PWUS, despite increasing recognition of these factors in broader nursing workforce literature. This absence is particularly striking given the ongoing impacts of the COVID-19 pandemic on nursing practice. Future research would benefit from longitudinal and mixed-methods designs that examine how individual attitudes intersect with occupational strain and organizational context.

While many studies linked nurses’ attitudes to patient experiences such as trust, engagement, and perceptions of care (Chan Carusone et al., 2019; McNeil et al., 2014; Strike et al., 2020), these relationships were typically inferred rather than empirically tested. This highlights a need for studies that more explicitly examine pathways between nurse attitudes and patient-level outcomes.

### 6.2 Mesolevel: hospital environment, intrapersonal interactions between nurses and PWUS

At the mesolevel, the reviewed literature consistently emphasized education and knowledge as central themes shaping nurses’ interactions with PWUS. Across studies, nurses reported perceived gaps in their preparation to care for patients with substance use difficulties, regardless of years of experience or prior training (Chozom et al., 2021; Menard-Kocik and Caine, 2021; Munoz et al., 2021; Shaw et al., 2016). Educational needs most frequently cited included pharmacology, withdrawal management, pain management, and contemporary substance use trends, particularly in the context of the opioid crisis (Costello and Thompson, 2015; Nusbaum and Farkash, 2022).

In addition to clinical knowledge, several studies highlighted stigma, bias, and communication challenges as key features of nurse and PWUS interactions. Interventions described in the literature, such as cultural safety education or inclusion of people with lived experience, were discussed as strategies explored within primary studies rather than as evidence-based solutions. Importantly, few studies evaluated the effectiveness or sustainability of these educational approaches, highlighting a methodological gap in outcome-focused research.

Gaps were also evident in prelicensure education. Multiple studies suggested that undergraduate nursing curricula provide limited content related to substance use and harm reduction (Nusbaum and Farkash, 2022; van Boekel et al., 2013). Gagnon et al. (2020) reported substantial variability and minimal instructional hours devoted to substance use across Canadian nursing programs. However, the literature largely lacked comparative or evaluative studies examining how curricular differences influence graduate preparedness or attitudes in practice.

Pain management emerged as a recurrent mesolevel challenge. Several studies described uncertainty, discomfort, and conflict in managing pain for PWUS, particularly in relation to opioid prescribing and assumptions of drug-seeking behavior (Horner et al., 2019; Strike et al., 2020). While some studies documented patient-reported experiences of pain dismissal and mistrust, few empirically examined interprofessional dynamics or institutional protocols shaping these interactions. This represents a significant gap, given the centrality of pain management to both patient outcomes and nurse moral distress.

### 6.3 Exolevel: hospital support, supporting nurses in caring for PWUS

At the exolevel, the literature described hospital policies, leadership support, and institutional culture as influential, yet inconsistently examined factors shaping nurses’ experiences caring for PWUS. Across studies, nurses frequently reported perceptions of inadequate organizational support, unclear policies, and limited access to addiction expertise within hospital settings (Antill Keener et al., 2023; Horner et al., 2019; Kratovil et al., 2023; Morgan, 2014; Nusbaum and Farkash, 2022).

Several studies discussed institutional policies related to substance use, security involvement, and harm reduction practices. However, most descriptions were descriptive or exploratory, and few studies assessed policy implementation processes, fidelity, or outcomes. Where harm reduction initiatives were examined, such as overdose prevention sites or integrated addiction services (Dogherty et al., 2022; Perera et al., 2022), the focus was typically on feasibility and acceptability rather than comparative effectiveness.

Methodologically, organizational-level studies were often context-specific and qualitative, limiting transferability. Additionally, the perspectives of nurses were frequently foregrounded, whereas leadership, policy-makers, and patients with lived experience were less consistently included. This suggests a need for multi-stakeholder research designs that examine organizational responses to substance use from multiple vantage points.

### 6.4 Macrolevel: societal influence, past and current political climates

At the macrolevel, harm reduction philosophy and broader sociopolitical contexts were intermittently referenced but rarely examined as primary analytic variables. Several studies situated their findings within a harm reduction paradigm, suggesting associations with reduced stigma and improved engagement in care (Fraimow-Wong et al., 2024; Goff et al., 2024; Perera et al., 2022). However, these associations were largely conceptual, as few studies empirically measured how macrolevel policy environments shape nurses’ attitudes or institutional practices.

Political and legislative contexts, such as shifting government support for harm reduction initiatives, were underrepresented in the literature, despite their potential influence on hospital policy and public discourse. For example, recent policy changes affecting supervised consumption services illustrate how political climates may shape both public attitudes and organizational responses to substance use. Yet, the reviewed studies rarely incorporated policy analysis or cross-jurisdictional comparisons. This gap limits understanding of how societal narratives, legislation, and funding priorities intersect with nursing practice at the bedside. Future research that explicitly integrates political context, health policy analysis, and cross-national comparisons would strengthen the evidence base and provide a more comprehensive understanding of nurses’ attitudes toward PWUS.

#### 6.4.1 Additional gaps noted in the literature

As mentioned previously, the majority of the articles had been published in the United States, with only a limited number of studies representing other countries. Further investigation into studies from diverse geographic locations could enrich the literature significantly, given the variations in substance use rates and health care systems across different regions. Many of the hospitals examined in these studies were situated in large urban centers, which may have led to an underrepresentation of patients from northern, rural, and remote areas. The unique challenges faced by these regions, such as geographic isolation, harsh weather conditions, complex population health issues, and economic hardship (Office of the Auditor General of Ontario, 2023), along with staffing shortages that often result in overextended nurses (Hall et al., 2016; Stemmer et al., 2022), may influence nurses’ attitudes in ways that are different from the attitudes of their urban counterparts.

Moreover, all of the studies had various methodological approaches and tools to measure and/or understand nurses’ attitudes. The most commonly used measurement tools in the quantitative studies, the AAPPQ (Cartwright, 1980) and the DDPPQ (Watson et al., 2007), were developed several decades ago and may no longer reflect current nursing attitudes accurately (Raynak et al., 2025). Given the significant advancements in the field of substance use, including the adoption of person-first language, harm reduction approaches, and evolving perspectives on addiction, these tools may not fully capture contemporary nursing attitudes toward substance use. The literature has suggested that psychometric properties of both the AAPPQ and DDPPQ (Mahmoud et al., 2021, 2023; Raynak et al., 2025; Terhorst et al., 2013) may be inconsistent, indicating the need for revisions or the development of more contemporary measures. Similarly, the qualitative literature varied in methodological approaches and in the structure and content of interviews, making it difficult to compare findings across studies or establish a cohesive understanding of nurses’ attitudes. Future research would benefit from the identification or development of assessment tools better aligned with current attitudes, practices, and the evolving landscape of substance use care.

Overall, this scoping review maps a growing but methodologically heterogeneous body of literature examining nurses’ attitudes toward PWUS in hospital settings. The SEM proved useful as an organizing framework to illustrate how attitudes are studied across individual, interpersonal, organizational, and societal levels. However, the literature remains heavily weighted toward micro- and mesolevel analyses, with comparatively limited empirical attention to organizational and macrolevel influences.

Consistent with the purpose of a scoping review, these findings highlight key gaps rather than definitive conclusions. Priority areas for future research include longitudinal studies of attitude development, evaluation of educational and organizational interventions, integration of patient and policy-maker perspectives, and explicit examination of political and policy contexts. Addressing these gaps will be essential to advancing a more comprehensive and methodologically robust understanding of nurses’ attitudes toward PWUS in hospital care.

### 6.5 Limitations

Several limitations should be noted. Many included studies provided limited methodological details, and the reliability and validity of questionnaires assessing nurses’ attitudes were often not reported. In some cases, questionnaire content was unclear, highlighting the lack of standardized approaches for measuring attitudes toward PWUS. Most studies captured only a single point in time, providing cross-sectional snapshots; longitudinal research could help clarify how nurses’ attitudes evolve and the factors associated with these changes. As with any scoping review, human error remains possible, including missed articles due to search or indexing limitations, delays in indexing, or unclear or incorrect titles and abstracts. This review also excluded grey literature and secondary research, which may have omitted relevant non–peer-reviewed evidence or synthesized findings, potentially limiting comprehensiveness. Future research could incorporate these sources to provide a broader understanding of the literature.

## 7 Conclusion

This scoping review identified frequent reporting of negative attitudes among nurses toward PWUS in the hospital setting. Using the SEM to contextualize the findings, key factors associated with these attitudes included gaps in knowledge and education surrounding substance use, concerns about pain management, and variations in hospital support. The review also revealed substantial gaps in the literature, including limited exploration of contextual factors such as workload and burnout, underrepresentation of regions outside the United States, particularly northern, rural, and remote areas and the lack of reliable, valid tools to measure nurses’ attitudes. These findings underscore the need for further research to better understand the extent, nature, and determinants of nurses’ attitudes toward PWUS and to inform the development of more rigorous measurement approaches. Expanding the evidence base in these areas is essential to advance knowledge and guide future investigations in this field.

## Funding

Andrea Raynak’s work is supported by the Canadian Institutes of Health Research through the Doctoral Student Research Award, 186,414

## CRediT authorship contribution statement

**Andrea Raynak:** Writing – review & editing, Writing – original draft, Visualization, Validation, Software, Resources, Project administration, Methodology, Investigation, Funding acquisition, Formal analysis, Data curation, Conceptualization. **France Paquet:** Writing – review & editing, Writing – original draft, Validation, Formal analysis, Data curation. **Amanda Bakke:** Writing – review & editing, Writing – original draft, Software, Formal analysis, Data curation. **Brianne Wood:** Writing – review & editing, Writing – original draft, Visualization, Validation, Supervision. **Michel Bédard:** Writing – review & editing, Writing – original draft, Validation, Supervision. **Christopher Mushquash:** Writing – review & editing, Writing – original draft, Validation, Supervision. **Debra Gold:** Writing – review & editing, Writing – original draft, Methodology. **Hunter Polonoski:** Writing – review & editing, Writing – original draft.

## Declaration of competing interest

The authors have nothing to declare.

## Figures and Tables

**Table:** 

SECTION	ITEM	PRISMA-ScR CHECKLIST ITEM	REPORTED ON PAGE#
TITLE			
Title	1	Identify the report as a scoping review.	1
ABSTRACT			
Structured summary	2	Provide a structured summary that includes (as applicable): background, objectives, eligibility criteria, sources of evidence, charting methods, results, and conclusions that relate to the review questions and objectives.	1-3
INTRODUCTION			
Rationale	3	Describe the rationale for the review in the context of what is already known. Explain why the review questions/objectives lend themselves to a scoping review approach.	5-6
Objectives	4	Provide an explicit statement of the questions and objectives being addressed with reference to their key elements (e.g., population or participants, concepts, and context) or other relevant key elements used to conceptualize the review questions and/or objectives.	6
METHODS			
Protocol and registration	5	Indicate whether a review protocol exists; state if and where it can be accessed (e.g., a Web address); and if available, provide registration information, including the registration number.	6
Eligibility criteria	6	Specify characteristics of the sources of evidence used as eligibility criteria (e.g., years considered, language, and publication status), and provide a rationale.	6-7
Information sources*	7	Describe all information sources in the search (e.g., databases with dates of coverage and contact with authors to identify additional sources), as well as the date the most recent search was executed.	7
Search	8	Present the full electronic search strategy for at least 1 database, including any limits such that it could be repeated.	47-48
Selection of sources of evidence†	9	State the process for selecting sources of evidence (i.e., screening and eligibility) included in the scoping review.	25-31
Data charting process‡	10	Describe the methods of charting data from the included sources of evidence (e.g., calibrated forms or forms tested by the team before their use, whether data charting was done independently or in duplicate) and any processes for obtaining and confirming data from investigators.	8
Data items	11	List and define all variables for which data were sought and any assumptions and simplifications made.	7-8
Critical appraisal of individual sources of evidence§	12	If done, provide a rationale for conducting a critical appraisal of included sources of evidence; describe the methods used and how this information was used in any data synthesis.	N/A
Synthesis of results	13	Describe the methods of handling and summarizing the data that were charted.	8

**Table:** 

SECTION	ITEM	PRISMA-ScR CHECKLIST ITEM	REPORTED ON PAGE#
RESULTS			
Selection of sources of evidence	14	Give numbers of sources of evidence screened, assessed for eligibility, and included in the review, with reasons for exclusions at each stage, ideally using a flow diagram.	8
Characteristics of sources of evidence	15	For each source of evidence, present characteristics for which data were charted and provide the citations.	9-16
Critical appraisal within sources of evidence	16	If done, present data on critical appraisal of included sources of evidence.	NA
Results of individual sources of evidence	17	For each included source of evidence, present the relevant data that were charted that relate to the review questions and objectives.	9-16
Synthesis of results	18	Summarize and/or present the charting results as they relate to the review questions and objectives.	9-16
DISCUSSION			
Summary of evidence	19	Summarize the main results, including an overview of concepts, themes, and types of evidence available; link to the review questions and objectives; and consider relevance to key groups.	16-21
Limitations	20	Discuss the limitations of the scoping review process.	21-22
Conclusions	21	Provide a general interpretation of the results with respect to the review questions and objectives, as well as potential implications and/or next steps.	22
FUNDING			
Funding	22	Describe sources of funding for the included sources of evidence, as well as sources of funding for the scoping review. Describe the role of the funders of the scoping review.	23

JBI = Joanna Briggs Institute; PRISMA-ScR = Preferred Reporting Items for Systematic reviews and Meta-Analyses extension for Scoping Reviews.

*Where *sources of evidence* (see second footnote) are compiled from, such as bibliographic databases, social media platforms, and Web sites.

† A more inclusive/heterogeneous term used to account for the different types of evidence or data sources (e.g., quantitative and/or qualitative research, expert opinion, policy documents) that may be eligible in a scoping review as opposed to only studies. This is a more inclusive term than “studies” and is aligned with the JBI guidance for scoping reviews.

‡ The process of systematically extracting information from the included sources of evidence. This is a more inclusive term than “data extraction” in a scoping review, as the process may not involve what is traditionally considered “data.”

§ The process of evaluating the included sources of evidence for their methodological quality and relevance before inclusion in a review, and determining whether and how this information will be used in the synthesis.

**Table:** 

Search #	Results
#1 nurse OR nurses OR registered nurses OR RPN OR LPN OR practical nurses	472,943
#2 Nurses[Mesh] OR licensed practical nurses[Mesh]	101,256
#3 #1 OR #2	472,943
#4 substance abuse OR substance use OR drug abuse OR drug addiction OR "drug use" OR	1,507,345
alcohol use OR alcohol addiction OR alcohol	
#5 Substance-Related Disorders[Mesh] OR "substance abuse detection[Mesh] OR "substance	327,065
abuse, intravenous[Mesh]	
#6 #4 OR #5	1,507,345
#7 nurses attitudes OR nurse perceptions OR nurse opinions OR nurse views	103,232
#8 Attitude of health personnel[Mesh]	173,393
#9 #7 OR #8	232,326
#10 #3 AND #6 AND #9	2,955
#11 Limited to 2014-2024 and in English	1,158

*Note.* PubMed does not have a Peer Review limiter

CINAHL (EBSCO), 2014-2024, English, Peer Reviewed

**Table:** 

Search #	Results
#1 nurse OR nurses OR registered nurses OR RPN OR LPN OR practical nurses	530,059
#2 MH Nurses OR MH registered nurses OR MH practical nurses	108,470
#3 #1 OR #2	530,059
#4 substance abuse OR substance use OR drug abuse OR drug addiction OR drug use OR alcohol 226,394 use or alcohol addiction OR alcohol	
#5 MH substance abuse OR MH substance use disorders	80,760
#6 #4 OR #5	226,394
#7 nurses attitudes OR nurse perceptions OR nurse opinions OR nurse views	44,257
#8 MH Nurse attitudes	41,372
#9 #7 OR #8	44,257
#10 #3 AND #6 AND #9	605
#11 #11 Limited to 2014-2024, peer reviewed and in English	237
Search #	Results
#1 nurse OR nurses OR registered nurses OR RPN OR LPN OR practical nurses	73,595
*#2* MAINSUBJECT.EXACT(Nurses)	30,567
*#3* #1 OR #2	73,595
*#4* substance abuse OR substance use OR drug abuse OR drug addiction OR drug use OR alcohol 324,304 use OR alcohol addiction OR alcohol	
*#5* MAINSUBJECT.EXACT("substance abuse and addiction measures") OR	11,105 MAINSUBJECT.EXACT("substance use disorder")
*#6* #4 OR #5	324,304
*#7* nurses attitudes OR nurse perceptions OR nurse opinions OR nurse views	33,548
*#8* MAINSUBJECT.EXACT(health personnel attitudes) OR MAINSUBJECT.EXACT(attitudes) 46,437	
*#9* #7 OR #8	72,002
*#10* #3 OR #6 OR #9	1,499
#11 Limited to 2014-2024, peer reviewed and in English	634

## Appendix B: Search Strategy

PubMed, 2014-2024, English, Peer Reviewed

## Appendix C Supplementary materials

Image, application 1

## References

[bib0001] Al-Awadhi A., Atawneh F., Alalyan M.Z.Y., Shahid A.A., Al-Alkhadhari S., Zahid M.A. (2017). Nurses’ attitude towards patients with mental illness in a general hospital in Kuwait. Saudi. J. Med. Med. Sci..

[bib0002] Allen, D., Brouwer, J., Dong, K., Dyer, D., Etches, N., Evans, L., Eyford, H., Gilani, F., Gill, H.S., Gill, S., Gurney, R., Hayer, L., Henderson, R., Hyshka, E., Lee, M., Leung, E., McCorkell, T., Meador, K., Morris, H., Woroniuk, A. (2020). *Guidance document on the management of substance use in acute care.* https://crismprairies.ca/management-of-substance-use-in-acute-care-settings-in-alberta-guidance-document/.

[bib0003] Antill Keener T., Tallerico J., Harvath R., Cartwright-Stroupe L., Shafique S., Piamjariyakul U. (2023). Nurses’ perception of caring for patients with substance use disorder. J. Addict. Nurs..

[bib0004] Aromataris, E., Lockwood, C., Porritt, K., Pilla, B., & Jordan, Z. (Eds.). (2024). *JBI manual for evidence synthesis.* 10.46658/JBIMES-24-01.

[bib0005] Atashzadeh-Shoorideh F., Monjazabi F., Fathollahzadeh E., Parastoo O. (2020). The obstacles to nurses being present with patients. Nurs. Open..

[bib0006] Babiarczyk B., Jonkisz D., Jaksz-Recmanik E. (2024). The attitudes of Polish nurses towards patients with alcohol-related problems and the subsequent impact on care delivery. Pielęgniarstwo XXI Wieku.

[bib0007] Bronfenbrenner U. (1979). The Ecology of Human development: Experiments by Nature and Design.

[bib0008] Cartwright A.K.J. (1980). The attitudes of helping agents towards the alcoholic client: the influence of experience, support, training, and self-esteem. Br. J. Addict..

[bib0009] Cazalis A., Lambert L., Auriacombe M. (2023). Stigmatization of people with addiction by health professionals: current knowledge. a scoping review. Drug Alcohol Depend. Rep..

[bib0010] Chan Carusone S., Guta A., Robinson S., Tan D.H., Cooper C., O’Leary B., De Prinse K., Cobb G., Upshur R., Strike C. (2019). Maybe if I stop the drugs, then maybe they’d care?”: hospital care experiences of people who use drugs. Harm. Reduct. J..

[bib0011] Chozom S., Neuhann F., Krahl W. (2021). Exploring the attitudes towards patients diagnosed with alcohol use disorder (AUD): A qualitative study of nurses at the national referral hospital, Bhutan. J. Nurs. Pract..

[bib0012] Christian N.J., Baysinger A., Bottner R., Cowley C., Nekolaichuk R., Owen P., Smith B., Sue K.L. (2025). Hospital-based stigma practices towards individuals with opioid use disorder: a qualitative study in Austin, Texas. Am. J. Med. Open..

[bib0013] Compton P., Aronowitz S.V., Klusaritz H., Anderson E. (2021). Acute pain and self-directed discharge among hospitalized patients with opioid-related diagnoses: a cohort study. Harm. Reduct. J..

[bib0014] Costello M., Thompson S. (2015). Preventing opioid misuse and potential abuse: the nurse's role in patient education. Pain Manag. Nurs. Off. J. Am. Soc. Pain Manag. Nurses..

[bib0016] de Vos A.J.B.M., de Kok E., Maassen S.M., Booy M., Weggelaar-Jansen A.M.J.W.M. (2024). Learning from a crisis: a qualitative study on how nurses reshaped their work environment during the COVID-19 pandemic. BMC. Nurs..

[bib0017] Dhanani L.Y., Franz B. (2021). Attitudes toward and experiences working with patients who misuse opioids among board certified physicians in Ohio. Subst. Abus..

[bib0018] Dogherty E., Patterson C., Gagnon M., Harrison S., Chase J., Boerstler J., Gibson J., Gill S., Nolan S., Ryan A. (2022). Implementation of a nurse-led overdose prevention site in a hospital setting: lessons learned from St. Paul’s Hospital, Vancouver, Canada. Harm. Reduct. J..

[bib0019] Dong K.A., Brouwer J., Johnston C., Hyshka E. (2020). Supervised consumption services for acute care hospital patients. Can. Med. Assoc. J..

[bib0020] Fraimow-Wong L., Martín M., Thomas L., Giuliano R., Nguyen O.K., Knight K., Suen L.W. (2024). Patient and staff perspectives on the impacts and challenges of hospital-based harm reduction. JAMA Netw. Open..

[bib0021] Gagnon M., Payne A., Denis-Lalonde D., Wilbur K., Pauly B. (2020). Substance use education in Canadian nursing programs: A student survey. J. Nurs. Educ..

[bib0022] Goff A., Lujan-Bear S., Titus H., Englander H. (2024). Integrating hospital-based harm reduction care-harnessing the nursing model. Subst. Use Addctn. J..

[bib0025] Grant K., Butterfield M., Bach P. (2025). Integrating chronic pain management into care for patients with opioid use disorder. CMAJ Can. Med. Assoc. J. J. Assoc. Med. Can..

[bib0027] Hall L.H., Johnson J., Watt I., Tsipa A., O’Connor D.B (2016). Healthcare staff wellbeing, burnout, and patient safety: a systematic review. PLoS. One.

[bib0028] Hakala T., Kylmae J., Paavilainen E., Koivunen M. (2020). The care of the patients with alcohol intoxication in the emergency department of a central hospital nurses’ skills, knowledge, and attitudes. J. Addict. Nurs..

[bib0029] Horner G., Daddona J., Burke D.J., Cullinane J., Skeer M., Wurcel A.G., Treloar C. (2019). You’re kind of at war with yourself as a nurse”: perspectives of inpatient nurses on treating people who present with a comorbid opioid use disorder. PLoS. One.

[bib0030] Hyde A., Johnson E., Bray C., Meier T., Carbonneau M., Spiers J., Tandon P. (2024). Understanding nurse perceptions of caring for patients with alcohol use disorder: a cross-sectional study. J. Addict. Nurs..

[bib0031] Hyshka E., Anderson-Baron J., Pugh A., Belle-Isle L., Hathaway A., Pauly B., Strike C., Asbridge M., Dell C., McBride K., Tupper K., Wild T.C. (2019). Principles, practice, and policy vacuums: policy actor views on provincial/territorial harm reduction policy in Canada. Int. J. Drug Policy..

[bib0034] Johansson L., Wiklund-Gustin L. (2016). The multifaceted vigilance: nurses’ experiences of caring encounters with patients suffering from substance use disorder. Scand. J. Caring Sci..

[bib0035] Kiepek N., Jones-Bonofiglio K., Freemantle S., Byerley-Vita M., Quaid K. (2021). Exploring care of hospital inpatients with substance involvement. Soc. Sci. Med..

[bib0036] Kratovil A., Schuler M.S., Vottero B.A., Aryal G. (2023). Original research: nurses’ self-assessed knowledge, attitudes, and educational needs regarding patients with substance use disorder. Am. J. Nurs..

[bib0037] Lake E.T., Pascale A., Warshawsky N.E., Smith J.G., Staiger D., Rogowski J.A. (2024). COVID-19 pandemic increases in nursing-sensitive quality indicators. Nurs. Res..

[bib0039] Lawrence S.A., Cicale C., Wharton T., Chapple R., Stewart C., Burg M.A. (2022). Empathy and attitudes about substance abuse among social work students, clinical social workers, & nurses. J. Soc. Work Pr. Addict..

[bib0042] Mahmoud K.F., Finnell D.S., Sereika S.M., Lindsay D., Schmitt K., Cipkala-Gaffin J., Puskar K.R., Mitchell A.M. (2021). Personal and professional attitudes associated with nurses’ motivation to work with patients with opioid use and opioid use-related problems. Subst. Abus..

[bib0043] Mahmoud K.F., Finnell D.S., Sereika S.M., Lindsay D., Cipkala-Gaffin J., Mitchell A.M. (2023). Factors associated with nurses’ motivation to provide care for patients with alcohol use and alcohol use-related problems. Subst. Abus..

[bib0044] Magnan E., Weyrich M., Miller M., Melnikow J., Moulin A., Servis M., Chadha P., Spivack S., Henry S.G. (2024). Stigma against patients with substance use disorders among health care professionals and trainees and Stigma-reducing interventions: a systematic review. Acad. Med. J. Assoc. Am. Med. Coll..

[bib0045] Mayer S., Langheimer V., Nolan S., Boyd J., Small W., McNeil R. (2023). Emergency department experiences of people who use drugs who left or were discharged from hospital against medical advice. PLoS. One.

[bib0046] Mclellan A.T. (2017). Substance misuse and substance use disorders: why do they matter in healthcare?. Trans. Am. Clin. Clim. Assoc..

[bib0047] McNeil R., Small W., Wood E., Kerr T. (2014). Hospitals as a risk environment: an ethno-epidemiological study of voluntary and involuntary discharge from hospital against medical advice among people who inject drugs. Soc. Sci. Med..

[bib0048] Menard-Kocik J., Caine V. (2021). Obstetrical nurses’ perspectives of pregnant women who use illicit substances and their provision of care: A thematic analysis. Can. J. Nurs. Res..

[bib0049] Merrick T.T., Louie E., Cleary M., Molloy L., Baillie A., Haber P., Morley K.C. (2022). A systematic review of the perceptions and attitudes of mental health nurses towards alcohol and other drug use in mental health clients. Int. J. Ment. Health Nurs..

[bib0050] Molina-Mula J., González-Trujillo A., Simonet-Bennassar M. (2018). Emergency and mental health nurses’ perceptions and attitudes towards alcoholics. Int. J. Environ. Res. Public Health.

[bib0051] Morgan B.D. (2014). Nursing attitudes toward patients with substance use disorders in pain. Pain. Manage Nurs..

[bib0052] Mulyani S., Suti Lasmani P., David Saifullah A., Fawadya A., Iffah A., Pramestya S. (2021). The attitudes of nurses in the hospital toward vulnerable people. Open. Access. Maced. J. Med. Sci..

[bib0053] Munoz K., Suchy C., Rutledge D.N. (2021). Knowledge and attitudes of maternity nurses and ancillary team members about substance addiction during pregnancy and postpartum. Am. J. Matern. Child Nurs..

[bib0054] Neville K., Roan N. (2014). Challenges in nursing practice: nurses’ perceptions in caring for hospitalized medical-surgical patients with substance abuse/dependence. J. Nurs. Adm..

[bib0055] Nusbaum L., Farkash M. (2022). Attitudes, perceptions, self-efficacy and knowledge levels of Israeli nurses in relation to opioid misuse: A cross-sectional survey. J. Nurs. Scholarsh..

[bib0056] Office of the Auditor General of Ontario (2023). https://www.auditor.on.ca/en/content/annualreports/arreports/en23/AR_hospitalsnorth_en23.pdf.

[bib0057] Ouzzani, M., Hammady, H., Fedorowicz, Z., & Elmagarmid, A. (2016). *Systematic reviews*, 5(210), 1–10. 10.1186/s13643-016-0384-4. PMC5139140 27919275

[bib0059] Perera R., Stephan L., Appa A., Giuliano R., Hoffman R., Lum P., Martin M. (2022). Meeting people where they are: implementing hospital-based substance use harm reduction. Harm. Reduct. J..

[bib0060] Peters M.D.J., Godfrey C.M., McInerney P., Baldini Soares C., Khalil H., Parker D., Aromataris E., Munn Z. (2020). Chapter 11: scoping reviews. JBI Manual For Evidence Synthesis.

[bib0061] Raynak A., Bédard M., Wood B., Mushquash C. (2025). Adapting the alcohol and alcohol problems perception questionnaire and the drug and drug problems perception questionnaire: A psychometric analysis of a person-centred approach. Drug Alcohol Depend. Rep..

[bib0062] Renbarger K.M., Phelps B., Brand J., Broadstreet A. (2021). Nurses’ descriptions of interactions when caring for women with perinatal substance use disorders and their infants. Nurs. Women Health.

[bib0063] Richelle L., Dramaix-Wilmet M., Roland M., Kacenelenbogen N. (2022). Factors influencing medical students’ attitudes towards substance use during pregnancy. BMC. Med. Educ..

[bib0064] Shaw M.R., Lederhos C., Haberman M., Howell D., Fleming S., Roll J. (2016). Nursesʼ perceptions of caring for childbearing women who misuse opioids. Am. J. Matern. Child Nurs..

[bib0065] Simon R., Snow R., Wakeman S. (2020). Understanding why patients with substance use disorders leave the hospital against medical advice: A qualitative study. Subst. Abus..

[bib0066] Stemmer R., Bassi E., Ezra S., Harvey C., Jojo N., Meyer G., Ozsaban A., Paterson C., Shifaza F., Turner M.B., Bail K. (2022). A systematic review: unfinished nursing care and the impact on the nurse outcomes of job satisfaction, burnout, intention-to-leave and turnover. J. Adv. Nurs..

[bib0067] Strike C., Robinson S., Guta A., Tan D.H., O’Leary B., Cooper C., Upshur R., Chan Carusone S. (2020). Illicit drug use while admitted to hospital: patient and health care provider perspectives. PLoS. One.

[bib0068] Sowicz T.J., Compton P., Matteliano D., Oliver J., Strobbe S., St Marie B., Turner H.N., Wilson M. (2022). Pain management and substance use disorders. Pain. Manag. Nurs. off. J. Am. Soc. Pain. Manag. Nurses..

[bib0069] Tan S.Y., Feng J.Y., Joyce C., Fisher J., Mostaghimi A. (2020). Association of hospital discharge against medical advice with readmission and In-hospital mortality. JAMa Netw. Open..

[bib0071] Temenos C., Koutlou A., Kyriakidou S., Galanaki S. (2024). Assessing stigma: health and social worker regard towards working with people using illicit drugs in Athens, Greece. Harm. Reduct. J..

[bib79] Terhorst L., Gotham H.J., Puskar K.R., Mitchell A.M., Talcott K.S., Braxter B., Hagle H., Fioravanti M., Woomer G.R. (2013). Confirming the factor structure of the alcohol and alcohol problems questionnaire (AAPPQ) in a sample of baccalaureate nursing students. Res. Nurs. Health.

[bib0073] Tricco A.C., Lillie E., Zarin W., O’Brien K.K., Colquhoun H., Levac D., Moher D., Peters M.D.J., Horsley T., Weeks L., Hempel S., Akl E.A., Chang C., McGowan J., Stewart L., Hartling L., Aldcroft A., Wilson M.G., Garritty C., Straus S.E. (2018). PRISMA extension for scoping reviews (PRISMA-ScR): checklist and explanation. Ann. Intern. Med..

[bib0074] van Boekel L.C., Brouwers E.P.M., Van Weeghel J., Garretsen H.F.L. (2013). Stigma among health professionals towards patients with substance use disorders and its consequences for healthcare delivery: systematic review. Drug Alcohol Depend..

[bib0075] Watson H., Maclaren W., Kerr S. (2007). Staff attitudes towards working with drug users: development of the drug problems perceptions questionnaire. Addiction.

[bib0076] Webster M., Tillson M., Annett J., Terrill D., Staton M. (2025). Healthcare utilization and perceived substance use-related stigma from healthcare workers among incarcerated women with opioid use disorder. Drug Alcohol Depend..

[bib0077] Wright, T., Hope, V., Ciccarone, D., Lewer, D., Scott, J., & Harris, M. (2020). Prevalence and severity of abscesses and cellulitis, and their associations with other health outcomes, in a community-based study of people who inject drugs in London, UK. PLoS. One, 15(7), e0235350. https://journals.plos.org/plosone/article/file?id=10.1371/journal.pone.0235350&type=printable. 10.1371/journal.pone.0235350 PMC7360031 32663203

[bib0078] Yaghmour S.M. (2022). Impact of settings and culture on nurses’ knowledge of and attitudes and perceptions towards people with dementia: an integrative literature review. Nurs. Open..

